# *Ab initio*-predicted micro-mechanical performance of refractory high-entropy alloys

**DOI:** 10.1038/srep12334

**Published:** 2015-07-22

**Authors:** Xiaoqing Li, Fuyang Tian, Stephan Schönecker, Jijun Zhao, Levente Vitos

**Affiliations:** 1 Department of Materials Science and Engineering, KTH - Royal Institute of Technology, 10044 Stockholm, Sweden; 2 Institute for Applied Physics, University of Science and Technology Beijing, 100083 Beijing, China; 3 Key Laboratory of Materials Modification by Laser, Ion and Electron Beams (Dalian University of Technology), Ministry of Education, 116024 Dalian, China; 4 Department of Physics and Astronomy, Division of Materials Theory, Uppsala University, Box 516, 75120 Uppsala, Sweden; 5 Research Institute for Solid State Physics and Optics, Wigner Research Center for Physics, P.O. Box 49, HU-1525 Budapest, Hungary

## Abstract

Recently developed high-entropy alloys (HEAs) consisting of multiple principal elements represent a new field of metallurgy and have demonstrated appealing properties for a wide range of applications. Using *ab initio* alloy theory, we reveal the alloying effect on the elastic properties and the ideal tensile strength (ITS) in the [001] direction of four body-centered cubic (bcc) refractory HEAs based on Zr, V, Ti, Nb, and Hf. We find that these HEAs show high elastic anisotropy and large positive Cauchy pressure, suggesting good extrinsic ductility. Starting from ZrNbHf, it is found that the ITS decreases with equimolar Ti addition. On the other hand, if both Ti and V are added to ZrNbHf, the ITS is enhanced by about 42%. An even more captivating effect is the ITS increase by about 170%, if Ti and V are substituted for Hf. The alloying effect on the ITS is explained by the *d*-band filling. An intrinsic brittle-to-ductile transition is found in terms of the failure mode under uniaxial tension. These investigations suggest that intrinsically ductile HEAs with high ideal strength can be achieved by controlling the proportion of group four elements to group five elements.

High-entropy alloys (HEAs) are potential engineering materials for demanding applications where exceptional damage tolerance, good wear, high oxidation and corrosion resistance is needed^1^,^2^. Although HEAs are composed of several equimolar or near equimolar elements, their solid solution phases often are face-centered cubic (fcc), hexagonal close packed (hcp) or bcc. As novel high-temperature structural and functional materials, HEAs consisting of refractory metallic elements (Ti, Zr, Hf, V, Nb, Ta, Cr, Mo, and W) are single bcc phase and have been in the focus of several experimental studies^3^,^4^,^5^,^6^,^7^,^8^,^9^,^10^,^11^,^12^.

High strength and good ductility are essential demands on the mechanical performance of structural materials. Some refractory HEAs with disordered bcc solid solution phase^5^,^8^, such as CrNbTiZr and CrNbTiVZr, were found to exhibit high compressive yield strength up to 1.3 GPa. The observed low ductility at room temperature limits, however, their potential application. The tensile strength of refractory HEAs has hardly been investigated. Hitherto, only Wu *et al*.^10^ reported a ~1 GPa ultimate tensile strength with a 14.9% plastic elongation for ZrTiNbHf, which were claimed to be the result of dislocation strengthening via dislocation multiplication.

While the extrinsic strength in real materials is controlled by the presence and propagation of dislocations, grain boundaries, cracks, and other microstructural defects, the ideal strength of a material is the strength at which a flawless crystal itself becomes unstable with respect to a homogeneous strain. This strength defines the intrinsic upper bound strength of the material connecting aspects of chemical bonding with mechanical properties of ideal lattices^13^. The mechanical failure mode of a perfect crystal under homogeneous deformation is employed to characterize the intrinsic ductility/brittleness of a material^14^. The ideal strength is involved in fracture theory and the nucleation of defects^13^,^15^,^16^,^17^ and has been accepted as a mechanical parameter for the design of high performance materials, especially for nano- or micro-size applications^13^,^18^,^19^,^20^,^21^,^22^. Today, no information on the intrinsic tensile properties of HEAs is available.

In this work, we reveal the compositional effect on the micro-mechanical properties of four HEAs in bcc phase composed of the refractory elements Ti, Zr, Hf, V, and Nb. The studied elastic constants and polycrystalline elastic moduli describe the mechanical properties in the small deformation region, and allow conclusions on extrinsic ductility/brittleness. The ideal tensile strength (ITS) displays the mechanical behavior for large elongations of ideal defect-free crystals where stress-strain relations are nonlinear. The ITS of the constituent refractory elemental solids in bcc structure (V, Nb, and Ta) is well understood^15^, the description of the ITS in multicomponent solid solutions is, however, rather limited. Recently, Li *et al*. determined the compositional dependence of bcc ternary V-based and binary Fe-based solid solutions from first-principles employing the coherent-potential approximation (CPA) to model chemical disorder^23^,^24^. Here we follow a similar computational procedure to establish the ITS of the present HEAs.

## Results and Discussion

The equilibrium volume (expressed here via the Wigner-Seitz radius) is the first physical parameter which can be used to assess the accuracy of the ab initio calculations. Table 1 compares the Wigner-Seitz radius from our calculation (*w*_*t*_) to those from experiments (*w*_exp_)^7^,^9^,^10^,^12^. For all HEAs considered here, the theoretical value is close to the experimental data. Furthermore, for all HEAs, the bcc structure is predicted to be energetically more stable than the fcc one (cf. also discussion below), in line with the observations.

The single-crystal elastic constants and polycrystalline elstic moduli are listed in Table 1. The calculated elastic constants demonstrate that all present refractory HEAs are elastically stable (*c*_44_ > 0, 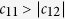, and *c*_11_ + 2*c*_12_ > 0). The structure stability of a cubic solid under tetragonal deformation is described by the tetragonal shear constants *c*′ (*c*′ = (*c*_11_ − *c*_12_)/2). Compared to ZrNbHf, titanium addition decreases *c*′, meaning that the ability of ZrTiNbHf to resist tetragonal shear decreases. However, if we substitute Hf in ZrNbHf by Ti and V, *c*′ becomes two times larger than that of ZrNbHf, which indicates that V strongly stiffens the bcc structure under tetragonal deformation. The Zener anisotropy ratio measures the anisotropy of elastic behavior, *A*_*Z*_ ≡ *c*_44_/*c*′ (for an isotropic cubic crystal, *A*_*Z*_ = 1). According to our results, *c*_44_ is almost constant for the investigated HEAs. The trend of the Zener anisotropy ratio is, hence, mostly dictated by the trend of *c*′. The *A*_*Z*_ data listed in Table 1 imply that all HEAs possess high elastic anisotropy. The anisotropy of ZrNbHf increases with adding Ti, however, decreases with introducing an equilmolar concentration of V simultaneously to Ti. The ZrVTiNb HEA possesses the largest Young’s modulus *Y*, and ZrTiNbHf exhibits the lowest one. The same behavior was found for the bulk modulus *B* and shear modulus *G*, however, their changes are smaller than *Y*.

The extrinsic ductility/brittlness of materials is connected with the magnitude of the *B*/*G* ratio and the sign of the Cauchy pressure (*c*_12_ − *c*_44_) by Pugh^25^ and Pettifor^26^, respectively. A simple physical picture behind the Pugh criterion is that *B* reflects the crystal cohesion and thus the resistance against cleavage, whereas *G* gives the opposition against dislocation movement. Polycrystalline materials are usually ductile when their *B*/*G* ratio is greater than 1.75 and when (*c*_12_ − *c*_44_) is positive; otherwise they are in the brittle regime. From Table 1, one can see that the *B*/*G* ratios are larger than 3.5, and (*c*_12_ − *c*_44_) are positive and large, suggesting that all investigated HEAs possess good extrinsic ductility.

**Figure 1:**
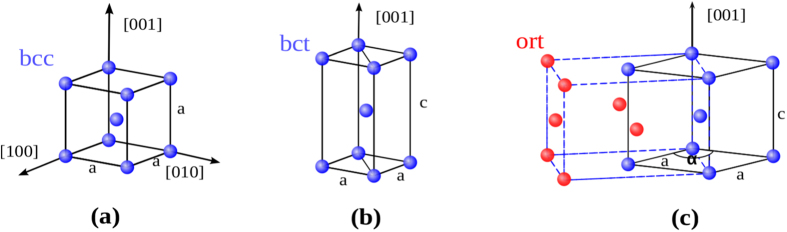
Schematic for the structures used to model the uniaxial strain in the [001] direction. Each atomic site is randomly occupied by equimolar elements. Unit cells are shown by solid lines for the unstrained lattice (bcc, panel **a**); the distorted tetragonal lattice (bct, panel **b**) and the distorted lattice after branching (panel **c**, *α* ≠ 90°). The latter can be described as face-centered orthorhombic lattice (ort, marked by dashed lines).

Turning in the following to the ITS of HEAs, we note that the [001] direction was already identified as the weakest direction of bcc systems^23^,^24^,^27^,^28^,^29^. Hence, here we consider the ITS of the four HEAs upon uniaxial loading along the [001] direction. The calculations followed the same quasi-static procedure as previously described^15^,^24^. Accordingly, the tensile stress *σ*(*ε*) was obtained from


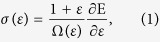


where E is the total energy per atom and Ω(*ε*) is the volume per atom at a given tensile strain, *ε*. At each value of the strain, the two unit cell lattice vectors perpendicular to the [001] direction were relaxed allowing for a possible symmetry lowering deformation relative to the initial body-centered tetragonal (bct) symmetry (see Fig. 1(b)), e.g., a branching to an orthorhombic (ort) symmetry (see Fig. 1(c)). The latter bifurcation was previously reported to occur for the refractory elements V, Nb, and Ta^15^. The first maximum on the stress-strain curve defines the ITS (*σ*_m_) with corresponding maximum strain (*ε*_m_).

**Figure 2:**
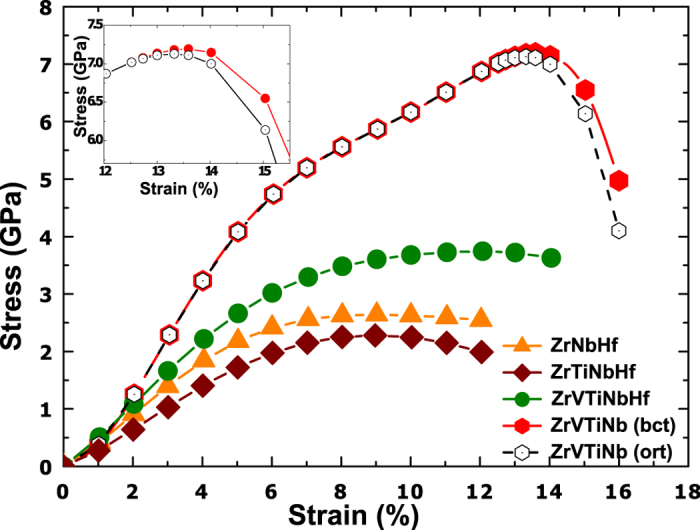
The tensile stress as a function of strain under uniaxial stress applied along the [001] direction for refractory HEAs. For ZrVTiNb, a branching from the tetragonal (bct) to the orthorhombic (ort) deformation path occurs at a strain of 12.7% < *ε*_m_ as shown more clearly in the inset; for larger strains, both the fully-relaxed orthorhombic and the constrained to bct stress-strain curves are plotted. Branching was not observed for the other alloys in the considering strain interval.

Figure 2 displays the tensile stress-strain relationships for bcc ZrNbHf, ZrVTiNb, ZrTiNbHf, and ZrVTiNbHf random alloys. The calculated ITSs and the corresponding maximum strains are listed in Table 1. We found that the ITS of the ternary ZrNbHf alloy amounts to 2.64 GPa, and slightly decreases to 2.28 GPa by adding the fourth element Ti. However, if we add Ti and V to ZrNbHf, the ITS is enhanced by nearly 42% with respect to the value of ZrNbHf. An even more pronounced impact of alloying on the ITS occurs if Hf in ZrNbHf is substituted with equiatomic Ti and V, which increases the ITS by about 170% (Table 1).

We found that three alloys, namely ZrNbHf, ZrTiNbHf, and ZrVTiNbHf, maintain bct geometry during tension along the [001] direction for strains up to slightly larger than *ε*_m_. Hence, these alloys fail by cleavage of the (001) planes and are representatives of bcc materials termed *intrinsically brittle* owing to their particular deformation and failure behaviors during tension^14^. In the case of the ZrVTiNb alloy, a symmetry lowering branching from bct to orthorhombic symmetry, caused by an elastic shear instability, occurs immediately before the maximum stress on the bct deformation path could be reached. This branching leads to a slight decrease of the ITS of ZrVTiNb with respect to the maximum value on the stress-strain curve assuming that branching is prohibited (the geometry was constrained to bct for these additional calculations). The constrained stress-strain curve and the stress-strain curve accounting for branching of ZrVTiNb are compared in Fig. 2. The aforementioned elastic instability can also be understood as an activated shear instability of the 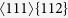 slip system (the resolved shear stress reaches the ideal shear stress under superimposed tension in [001] direction)^30^. The resolved shear stress for the 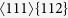 slip system at the instability is 3.5 GPa for ZrVTiNb, which is a good estimate of its ideal shear strength (*τ*_m_) for the same slip system without superimposed tension^29^. The estimated reduced shear strength, given by 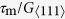, 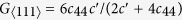, amounts to 0.1 in agreement with other bcc systems^15^,^31^. Owing to this particular failure behavior, the ZrVTiNb alloy is referred to as being *intrinsically ductile*, which represents the preferred failure mode over the intrinsically brittle one^14^. Thus, ZrVTiNbHf and ZrVTiNb display an intrinsic brittle-to-ductile transition in terms of their intrinsic failure mode which is altered upon changing the composition of the alloy, *i.e*., removal of Hf.

In order to understand the trend of the ITS for the present HEAs, we first realize that the alloy with the highest ITS (ZrVTiNb) contains two elements from the fourth group and two from the fifth group, which can be denoted by the compositional ratio 2:2. In contrast, ZrTiNbHf, possessing the lowest ITS, has a composition dominated by elements from group four (ratio 3:1). The alloy with moderate ITS (ZrVTiNbHf) consists of totally five elements with ratio 3:2. This simple analysis indicates that the ITSs of refractory HEAs are determined by the ratio of constituent transition metal elements belonging to the fourth group and fifth group. Obviously, the smaller is the ratio the higher is the ITS. It further suggests that the ITS of refractory HEAs could be tuned by varying the total number of equimolar transition metals elements. Both observations are explicated in the following.

**Figure 3:**
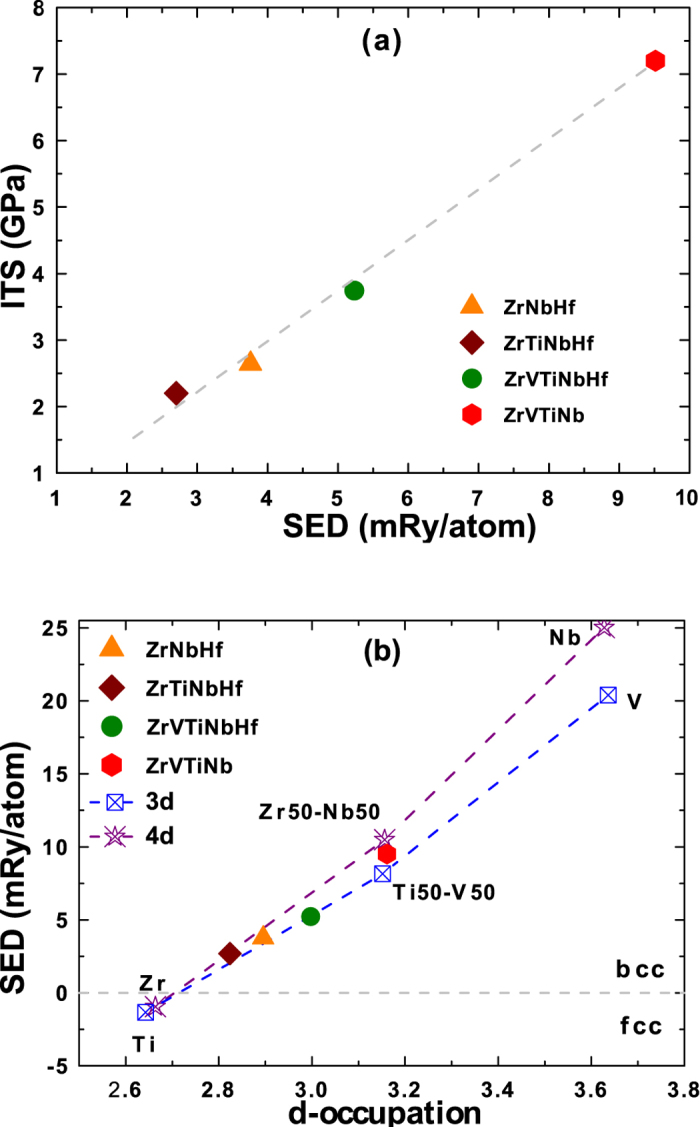
(**a**) The correlation between the ITS and the fcc-bcc SED for the HEAs. (**b**) The correlation between the fcc-bcc SED and the average valence *d*-band filling for the HEAs (solid symbols), group 4 and 5, 3*d*- ad 4*d* transition metals and their random binaries. Dashed lines guide the eye.

It is well known that the cohesive properties of the three transition metal series are mainly determined by the *d*-electrons, and the observed sequence of stable crystal structures as well as trends of cohesive properties originate from the gradual filling of the *d*-band^32^,^33^,^34^,^35^. For a more elaborate analysis of the ITSs, we show in the following that the ITS of the present HEAs can be understood by band-filling arguments. To this end, we employ the structural energy difference (SED) model of the ITS recently suggested for nonmagnetic V-Ti-Cr solid solutions^23^ Accordingly, the ITS of an intrinsically brittle, bcc stable material is correlated with the fcc-bcc SED, *σ*_m_ ∝ Δ*E*^SED^ ≡ *E*_fcc_ − *E*_bcc_. In this model, the energy difference is computed at constant volume equal to the bcc equilibrium volume. Although ZrVTiNb was found to be intrinsically ductile, we also apply the SED model to ZrVTiNb, since its ITS is only slightly lowered by branching as discussed above. Figure 3(a) shows a very strong correlation between the ITS and the SED for the presently investigated HEAs, i.e., higher fcc-bcc SED corresponds to higher ITS. Encouraged by the strong correlation between the ITS and the SED, we plotted the determined Δ*E*^SED^s for the present HEAs as a function of the *d*-band filling in Fig. 3(b). The *d*-band filling of alloys was obtained by averaging the number of valence *d*-electrons of the constituent in the alloy. The data for the HEAs in Fig. 3(b) clearly reveals that the SED is a monotonic, nearly linear function of the average *d*-occupation.

Electronic structure theory showed that the fcc-bcc SED itself follows a regular pattern for all three transition metal series, as a function of the number of valence *d*-electrons^32^,^36^. For a *d*-band filling corresponding to bulk Ti (Zr, Hf), Δ*E*^SED^ < 0, but for a *d*-band filling corresponding to bulk V (Nb, Ta), Δ*E*^SED^ > 0. This implies that Δ*E*^SED^ increases as the number of 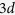 (4*d*, 5*d*) electrons increases and changes sign. Both properties of Δ*E*^SED^ are confirmed by the present theory and are shown for the 3*d* (Ti, V) and 4*d* (Zr, Nb) elements and their equimolar random solid solutions (Ti-V, Zr-Nb) in Fig. 3(b). Surprisingly, the SED of the multicomponent HEAs follows very closely the trend of the SED inherent to the 3*d* or 4*d* transition metal series (for the 5*d* series, see Refs 32,36). This close correlation confirms the fact that the SED of the present refractory HEAs is mainly determined by their average *d*-occupation number. Combining the above discussion and the data in Fig. 3, our results show that the ITS of the present refractory HEA is correlated with their average *d*-occupation, i.e., the higher the *d*-occupation, the larger the ITS. We notice that *c*′ increases monotonically with the ITS, and consequently with the average *d*-occupation number. No similar correlation was found in the case of magnetic bcc alloys^24^.

It is important in this context to realize that, although Ti, Zr, and Hf are not stable in the bcc structure (at ambient conditions), the HEAs composed to a major part of elements from the fourth group are, in fact, bcc stable. This in turn suggests, that there is great flexibility to tune the ITS of refractory HEA by varying composition and number of constituent elements as illustrated by the present work. The highest values of the ITS are obtained for HEA containing a large amount of group five elements due to their high intrinsic ITS (approximately 12–14 GPa^15^). The benefit of forming HEA by alloying group four elements to group five elements is a wide stability range of the bcc phase (in terms of the average *d*-occupation number, see Fig. 3(b)) at the cost of lowering the ITS. The ZrVTiNb alloy with *d*-occupation approximately equal to 3.15 is intrinsically ductile as are the elements V, Nb, and Ta with larger *d*-occupation approximately equal to 3.6. Given the present finding that the *d*-occupation is the controlling variable for the ITS, one might expect, that HEAs with *d*-band fillings larger than ~3.2 are also intrinsically ductile and reach ITS values that approach those of the group five elements, as the *d*-occupation approaches ~3.6.

## Conclusion

We have employed *ab intio* alloy theory to investigate the equilibrium volume, elastic properties, and the ideal tensile strength for the single bcc-phase HEAs: ZrNbHf, ZrVTiNb, ZrTiNbHf, and ZrVTiNbHf. The predicted equilibrium volumes are consistent with the available experimental data. All investigated HEAs exhibit good extrinsic ductility shown by the large *B*/*G* ratio and the positive Cauchy pressure (*c*_12_ − *c*_44_). For the HEAs, we also found that the ideal tensile strengths increase with decreasing the ratio of the fourth group elements to fifth group elements. The alloying effect on the ideal tensile strength of HEAs is explained by the *d*-band filling. In light of the recent attempts connecting the intrinsic materials properties to the extrinsic ductility^37^, the present results offer a guideline to design ductile refractory HEAs with tailored strength level.

## Methods

The present calculations were performed with the exact muffin-tin orbitals (EMTO) method in combination with the CPA^38^,^39^,^40^, which were previously employed to study the structure, mechanical properties, and phase transformation of other HEAs^41^,^42^. In the present application, the EMTO-CPA calculations were solved within the frozen-core scheme using the local-density-approximation (LDA) exchange-correlation density functional^43^. The Green’s function was calculated for 24 complex energy points around the valence states. The basis set included *s*, *p*, *d*, and *f* states. Brillouin zone integrations were performed on a 27 × 27 × 27 *k*-points mesh. The electrostatic correction to the single-site CPA was described using the screened impurity model. The 5*d*, 6*s* and 4*f* electrons were treated as valence states for Hf. The technical details of the elastic constants/modulus calculations are identical to those described in Ref. 41

## Additional Information

**How to cite this article**: Li, X. *et al*. Ab *initio*-predicted micro-mechanical performance of refractory high-entropy alloys. *Sci. Rep*. **5**, 12334; doi: 10.1038/srep12334 (2015).

## Figures and Tables

**Table 1:** Theoretical (EMTO-CPA) bulk parameters calculated for the present HEAs.

HEA	*w*_*t*_	*w*_exp_	*B*	*c*_11_	*c*_12_	*c*_44_	*c*′	*c*_12_–*c*_44_	*A*_*Z*_	*v*	*G*	*Y*	*B*/*G*	*σ*_m_	*ε*_m_
ZrNbHf	3.319	3.240	140.7	162.7	129.6	55.9	16.5	73.7	3.385	0.387	34.4	95.4	4.09	2.64	9.0
ZrVTiNb	3.089	3.094	149.4	192.8	127.9	51.7	32.5	76.2	1.595	0.369	42.3	117.5	3.53	7.13	13.3
ZrTiNbHf	3.265	3.274	136.7	154.3	127.9	56.7	13.2	71.1	4.286	0.392	31.9	88.9	4.29	2.28	8.9
ZrVTiNbHf	3.190	3.145	143.9	170.2	130.8	51.3	19.7	79.5	2.606	0.388	35.0	97.1	4.11	3.74	12.1

Listed are the equilibrium Wigner-Seitz radius *w*_*t*_ (in Bohr), elastic constants *c*_11_, *c*_12_, *c*_44_, *c*′, and Cauchy pressure (*c*_12_–*c*_44_) (in GPa), Zener anisotropy ratio *A*_*Z*_; polycrystalline elastic moduli *B*, *G*, *Y* (in GPa), and Poisson’s ratio *v*, *B*/*G* ratio; the ideal tensile strength *σ*_m_ (in GPa) in the [001] direction and the corresponding strain *ε*_m_ (in %). The available experimental Wigner-Seitz radii are also shown^7^,^9^,^10^,^12^.
